# Synergism of X-rays and bleomycin on Ehrlich ascites tumour cells.

**DOI:** 10.1038/bjc.1977.205

**Published:** 1977-09

**Authors:** Y. Takabe, T. Miyamoto, M. Watanabe, T. Terasima


*Br. J. Cancer* (1977) **36**, 391

***Short Communication***

### SYNERGISM OF X-RAYS AND BLEOMYCIN ON EHRLICH ASCITES TUMOUR CELLS

Y. TAKABE*, T. MIYAMOTO*, M. WATANABE* AND T. TERASIMA†

*From the *First Department of Medicine, Chiba University School of Medicine, Inohana, Chiba-si* 280 *and †Division of Physiology and Pathology, National Institute of Radiological Sciences, Anagawa*-4, *Chiba-si* 280, *Japan*

Received 6 April 1977 Accepted 13 May 1977

COMBINED use of X-rays and bleomycin was initially proposed by Jørgensen (1972). The demonstration of synergism attracted much attention from clinicians as well as basic researchers. The question arose whether the effect can be attributed to a tissue mechanism or to cellular potentiation. In the mean time, several investigations have been carried out to see whether the combination is synergistic in bacterial and mammalian cell systems (Bleehen, Gillies and Twentyman, 1974; Terasima, Takabe and Yasukawa, 1975; Bistrović, Maricić and Kolarić, 1976). Although the experimental results were not consistent among the cell lines used, the synergism was found to a slight or moderate extent.

The potentiation presented here, with Ehrlich ascites tumour cells treated with a combination of X-rays and bleomycin, may involve an interaction of different types of damage and repair induced by both agents.

Experiments were carried out with Ehrlich ascites tumour cells grown in 4-week-old male mice ICR/JCL, weighing 20–25 g (CLEA JAPAN Inc., Tokyo). By inoculating $10^6$ tumour cells i.p., the early plateau phase of growth was reached on the 7th day, when all the experiments were initiated.

To assay survival of tumour cells, they were removed from the abdominal cavity and suspended in F10 medium (Ham, 1963) with 10% calf serum. The cells were plated out in triplicate dishes of soft agar after they had been counted with electronic counter (Coulter, Model B) and diluted appropriately with F10 medium. The agar colony assay has been described in detail by Takabe *et al.* (1977). The plating efficiency (PE) for untreated cells was usually 40–90%. To estimate the surviving fraction, a portion of ascites was removed from each animal just before the initial treatment with either agent and the PE of untreated tumour cells was assayed for individual mice. Thus, surviving fraction was expressed as the PE of tumour cells treated with agent(s) divided by the PE of untreated cells from the same animal.

Bleomycin complex (Lot No. F100S41, Nippon Kayaku Co., Ltd, Tokyo) was dissolved in distilled water and diluted in F10 medium at the time of experiments. Approximately 0·3 ml of drug solution was injected through an s.c. route in the back of the mouse. The volume varied slightly with the body weight of individual tumour-bearing mice. All irradiations were given as whole-body doses to unanaesthetized mice by X-ray generator (Sinai, Shimazu Rad. Instr. Co., Ltd, Kyoto) operated at 200 KVp, 20 mA with added filtration (HVL: 1·2 mm Cu). Tumour-bearing mice housed in individual spaces of a round, lucite box were irradiated on a turntable at the dose rate of 80 rad/min.

Mice bearing tumour cells were either

Address for correspondence: T. Terasima, National Institute of Radiological Sciences, Anagawa-4, Chiba-si, Japan 280.

treated with a single dose of 0·1 mg/kg bleomycin or irradiated with a single dose of 400 rad X-rays. At times indicated in Fig. 1A, ascites fluid was removed repeatedly from the abdominal cavity of the same animal and survival of tumour cells was assayed. Starting from 1 h after administration of bleomycin, the survival increased quickly with time and finally levelled off at 5 h (open circles), whereas the X-ray survival increased rather slowly, and almost attained a plateau at about 7 h (closed circles). The survivals, measured as a ratio of 7-h value to the value of initial determination (surviving fraction ratio, after Evans *et al.*, 1974) were 1·7 and 1·5 respectively. The number of ascites tumour cells was determined following treatment with either single agent. The result showed that cells did not resume their proliferation within the 7-h observation period. Therefore, the observed enhancement of survival represents the repair of damage which was potentially lethal and reparable only when cells were in the abdominal cavity (Belli, Dicus and Nagle, 1970; Little *et al.*, 1973).

Survivals were also determined with time after both agents were given simultaneously, and are shown in Fig. 1A (open triangles). The surviving fraction ratio was about 2·5 during the first 7 h, indicating that repair took place as found after administration of either single agent. The survival level was consistently lower throughout the 7-h period than the level expected when damage produced by each agent was independently repaired (broken line).

A similar experiment was carried out with single doses of 30 mg/kg bleomycin and of 1000 rad X-rays, as shown in Fig. 1B. The surviving fraction ratios given by each agent were much greater than those in the preceding experiment, *i.e.*, 4·1 for bleomycin and 4·2 for X-ray. In this case, the survivals after simultaneous administration of agents (open triangles) were obviously lower than the level expected from independent (or additive) effects of each agent (broken line). This finding indicates that bleomycin potentiated the effect of X-rays, either by interfering with the repair process of, or by

FIG. 1.—Change in survival of Ehrlich ascites tumour cells after separate or simultaneous administration of bleomycin and X-rays. A: open circles—0·1 mg/kg bleomycin alone; closed circles—400 rad X-ray alone; triangles—simultaneous administration. B: open circles—30 mg/kg bleomycin alone; closed circles—1000 rad X-rays alone; triangles—simultaneous administration. Broken line was given by a product of surviving fractions obtained at various times after the single treatment with bleomycin (open circles) and X-rays (closed circles). It represents survival level which would be expected from additive effect of both agents. Survival value with error bar denotes mean ± s.d. of 4 separate determinations.

interacting with, damage induced by X-rays. In the experiment with lower doses as shown in Fig. 1A, the extent of potentiation was 20–25% of survival expected for the additive effect, whereas it was 50–85% in the case of higher doses (Fig. 1B). This may imply that the more the amount of damage, the greater the potentiation.

Fig. 2 shows the result of experiments in which bleomycin was given to mice at various times after X-irradiation. A group of tumour-bearing mice was irradiated with 400 rad at zero time. Then a single dose of bleomycin (0·1 mg/kg) was administered to each mouse at times indicated. A tumour-bearing mouse was used for a single determination. Survival of tumour cells was determined exactly 1 h after injection of bleomycin and was plotted at the time of drug administration (closed circles). Increase of survival after exposure to X-rays alone (open circles) is taken from Fig. 1A. If a single dose of bleomycin (surviving fraction 0·54 in Fig. 1A) exerted only an independent effect, the expected survival level would be given as a product of surviving fraction of both agents, as shown by the broken line. The results showed that the survival determined was clearly lower than the expected value at zero time, then increased with time and reached the level of independent effect after about 3 h.

The next experiments (Fig. 3) were carried out in a similar fashion to the preceding ones, except that bleomycin was injected at various times *before* X-ray exposure. A group of tumour-bearing mice was treated with a single 0·1-mg/kg dose of bleomycin at zero time. From 1 h on, each mouse was irradiated with 400 rad at

FIG. 2.—Effect of post-irradiation exposure to bleomycin on survival of Ehrlich ascites tumour cells. Straight arrows indicate times when bleomycin (BLM) was introduced. Closed symbols: survival for X-rays followed by BLM. Bar denotes the range of determinations. Open symbols: survival for X-rays alone (from Fig. 1A). Broken line represents the additive effect obtained as a product of surviving fraction at various times after exposure to X-rays alone and surviving fraction assayed 1 h after BLM (*i.e.* 0·54 from Fig. 1A).

FIG. 3.—Effect of pre-irradiation exposure to bleomycin on survival of Ehrlich ascites tumour cells. Closed symbols: survival for X-ray with BLM pretreatment. Bar denotes the range of determinations. Open symbols: survival for BLM alone (from Fig. 1A). Broken line represents the expected level of additive effect, obtained as a product of surviving fraction at various times after administration of BLM and surviving fraction after 400 rad X-rays (*i.e.* 0·495 from Fig. 1A).

specified times. Immediately afterwards, the ascites fluid was removed and the surviving fraction of tumour cells assayed. Therefore, one animal served for a single determination. Enhancement of survival after administration of bleomycin alone (open circles) is as shown in Fig. 1A. If the effect of each agent were independent, the expected survival would follow the broken line, which is a product of survivals of both agents. Experimental points were obviously lower than the broken line over the first 3 h, and then became close to the level of independent or additive effect. These results revealed that (i) more than additive effect can be obtained only when the interval between two agents was less than 3 h, whatever the order of administration, and (ii) the potentiation is greater if two agents are given at closer intervals.

Recently, the induction and repair of potentially lethal damage was demonstrated in tumour cells treated with bleomycin (Takabe *et al.*, 1974; Barranco, Novak and Humphrey, 1975; Twentyman and Bleehen, 1975). Nevertheless, the antibiotic does not induce sublethal damage and repair, as the simple exponential nature of the survival curve suggested (Terasima *et al.*, 1972; Barranco *et al.*, 1975).

To effect maximal sterilization of tumour cells treated with an agent inducing potentially lethal damage, the repair must be controlled, either by inhibiting enzymatic repair reactions or by fixing potentially lethal damage *per se*. The potentiation found after the simultaneous administration of both agents (Fig. 1) suggests that at least portions of the damage, either potentially lethal or sublethal, induced by the two agents interacted each other and were converted to lethal damage. The limited period of potentiation found in Fig. 2 may suggest that the potentiation involves X-ray-induced sublethal damage, the repair of which normally completes approximately 3 h after X-irradiation. Similarly, the combined effect of bleomycin administered before X-rays may be also related to repair of bleomycin-induced damage (Fig. 3). The fact that both agents induce reparable damage of cellular DNA (Tsuboi and Terasima, 1970; Terasima, Yasukawa and Umezawa, 1970; Fujiwara and Kondo, 1973; Saito and Andoh, 1973) may be a basis for part of the potentiation.

The observed interaction between damages due to X-ray and bleomycin may provide practicable means to control repair. Using transplantable mouse tumour, Jørgensen (1972) demonstrated that simultaneous administration resulted in greater reduction in tumour weight than alternating administration. The result may be understood on the basis of the damage-interaction hypothesis suggested here.

The present study was supported by a grant from the Japanese Ministry of Education under the auspices of Prof. M. Sakka, Tohoku University.

### REFERENCES

Barranco, S. C., Novak, J. K. & Humphrey, R. M. (1975) Studies on Recovery from Chemically Induced Damage in Mammalian Cells. *Cancer Res.*, **35**, 1194.

Belli, J. A., Dicus, G. J. & Nagle, W. (1970) Repair of Radiation Damage as a Factor in Preoperative Radiation Therapy. *Front. Radiat. Ther. Onc.*, **5**, 40.

Bistrović, M., Maricić, Z. & Kolarić, K. (1976) Interaction of Bleomycin and Radiation in Combined Treatment of Mouse L Cells. *Int. J. Cancer*, **18**, 540.

Bleehen, N. M., Gillies, N. E. & Twentyman, P. R. (1974) The Effect of Bleomycin and Radiation in Combination on Bacteria and Mammalian Cells in Culture. *Br. J. Radiol.*, **47**, 346.

Evans, R. G., Bagshaw, M. A., Gordon, L. F., Kurkjian, S. D. & Hahn, G. M. (1974) Modification of Recovery from Potentially Lethal X-ray Damage in Plateau Phase Chinese Hamster Cells. *Radiat. Res.* **59**, 597.

Fujiwara, Y. & Kondo, T. (1973) Strand Scission of HeLa Cell Deoxyribonucleic Acid by Bleomycin *In vitro* and *In vivo*. *Biochem. Pharmacol.*, **22**, 323.

Ham, R. G. (1963) An Improved Nutrient Solution for Diploid Chinese Hamster and Human Cell Lines. *Expl Cell Res.*, **29**, 515.

Jørgensen, S. J. (1972) Time-dose Relationships in Combined Bleomycin Treatment and Radiotherapy. *Eur. J. Cancer*, **8**, 93.

Little, J. B., Hahn, G. M., Frindel, E. & Tubiana, M. (1973) Repair of Potentially Lethal Radiation Damage *In vitro* and *In vivo*. *Radiology*, **106**, 689.

Saito, M. & Andoh, T. (1973) Breakage of a DNA-Protein Complex Induced by Bleomycin and their Repair in Cultured Mouse Fibroblasts. *Cancer Res.*, **33**, 1696.

TAKABE, Y., WATANABE, M., MIYAMOTO, T. & TERASIMA, T. (1974) Demonstration of Repair of Potentially Lethal Damage in Plateau Phase Cells of Ehrlich Ascites Tumor after Exposure to Bleomycin. *Gann*, **65**, 559.

TAKABE, Y., MIYAMOTO, T., WATANABE, M. & TERASIMA, T. (1977) Bleomycin: Mammalian Cell Lethality and Cellular Basis of Optimal Schedule. *J. natn. Cancer Inst.* (in press).

TERASIMA, T., YASUKAWA, M. & UMEZAWA, H. (1970) Breaks and Rejoining of DNA in Cultured Mammalian Cells Treated with Bleomycin. *Gann*, **61**, 513.

TERASIMA, T., TAKABE, Y., KATSUMATA, Y., WATANABE, M. & UMEZAWA, H. (1972) Effect of Bleomycin on Mammalian Cell Survival. *J. natn. Cancer Inst.*, **49**, 1093.

TERASIMA, T., TAKABE, Y. & YASUKAWA, M. (1975) Combined Effect of X-rays and Bleomycin on Cultured Mammalian Cells. *Gann*, **66**, 701.

TSUBOI, A. & TERASIMA, T. (1970) Rejoining of Single Breaks of DNA Induced by X-rays in Mammalian Cells: Effects of Metabolic Inhibitors. *Molec. gen. Genetics*, **108**, 117.

TWENTYMAN, P. R. & BLEEHEN, N. M. (1975) Studies of Potentially Lethal Damage in EMT6 Mouse Tumour Cells Treated with Bleomycin either *In vitro* or *In vivo*. *Br. J. Cancer*, **32**, 491.


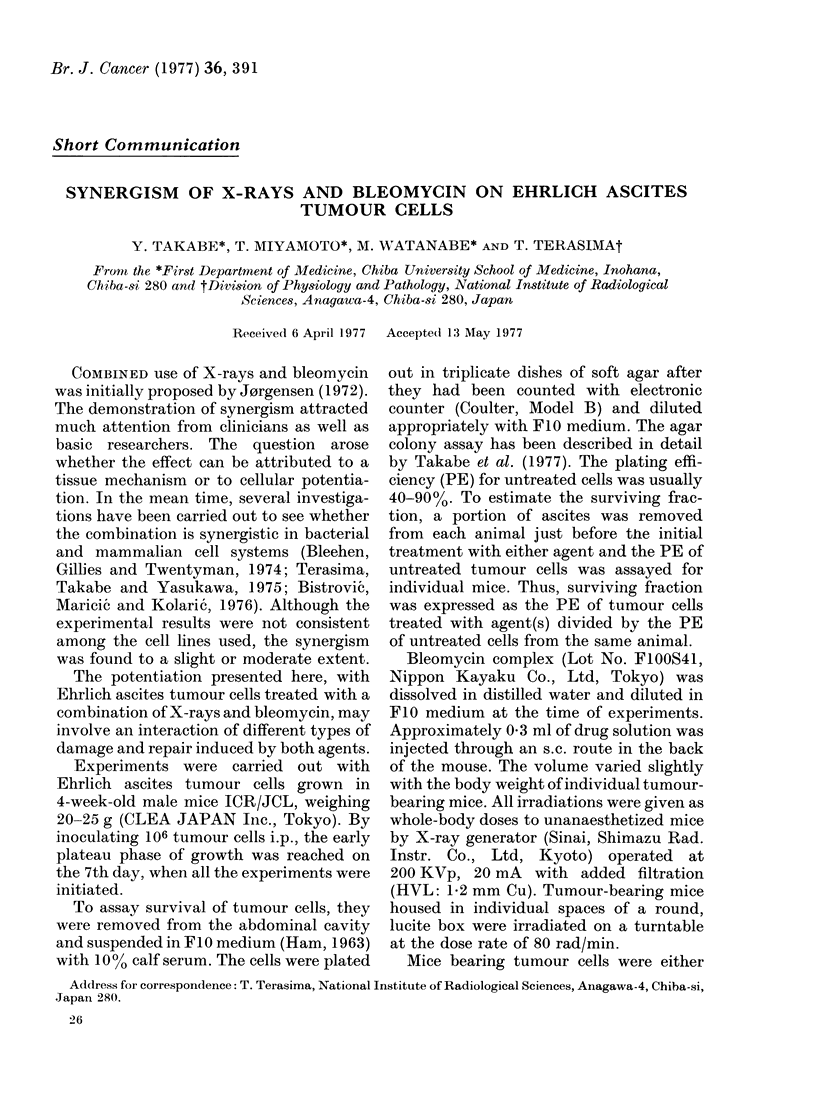

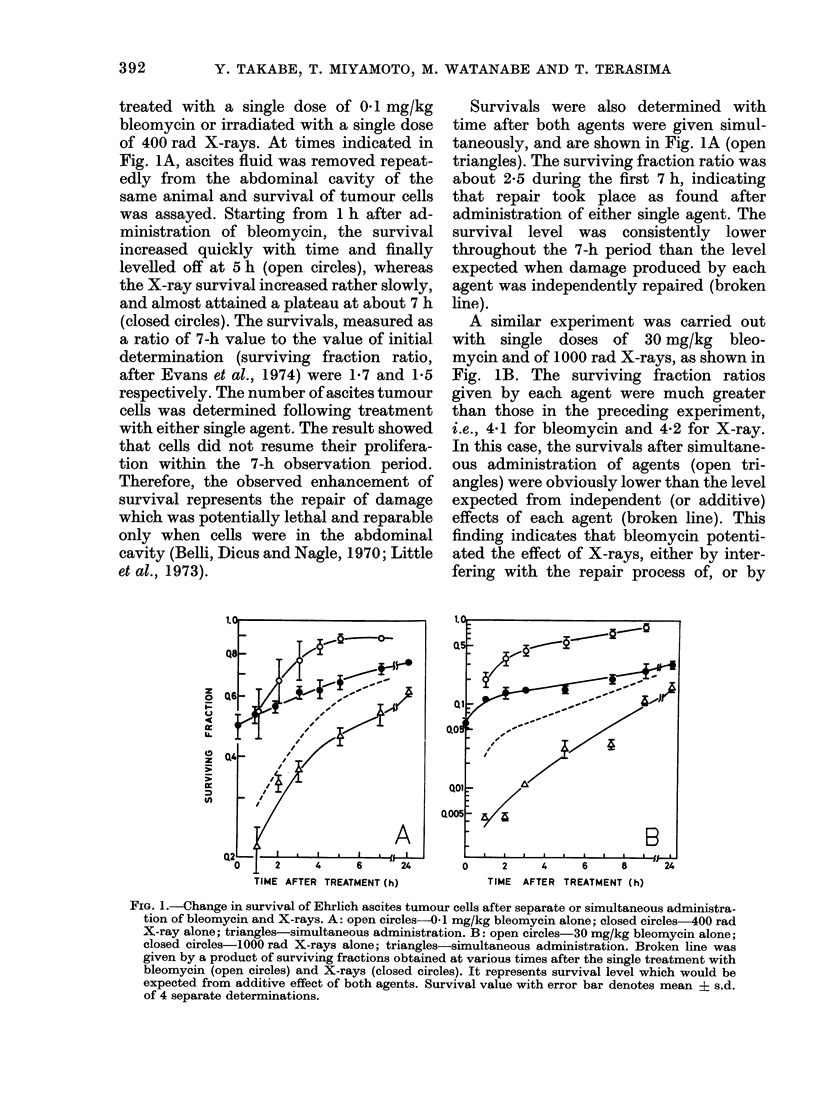

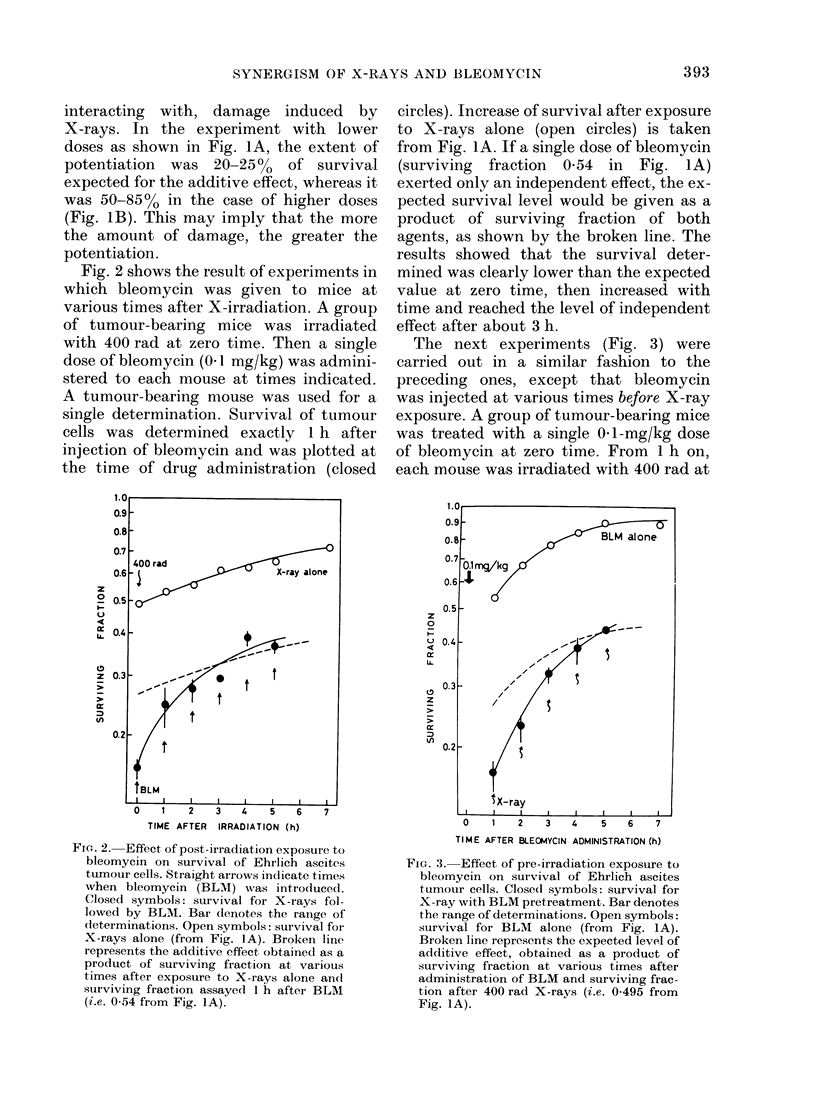

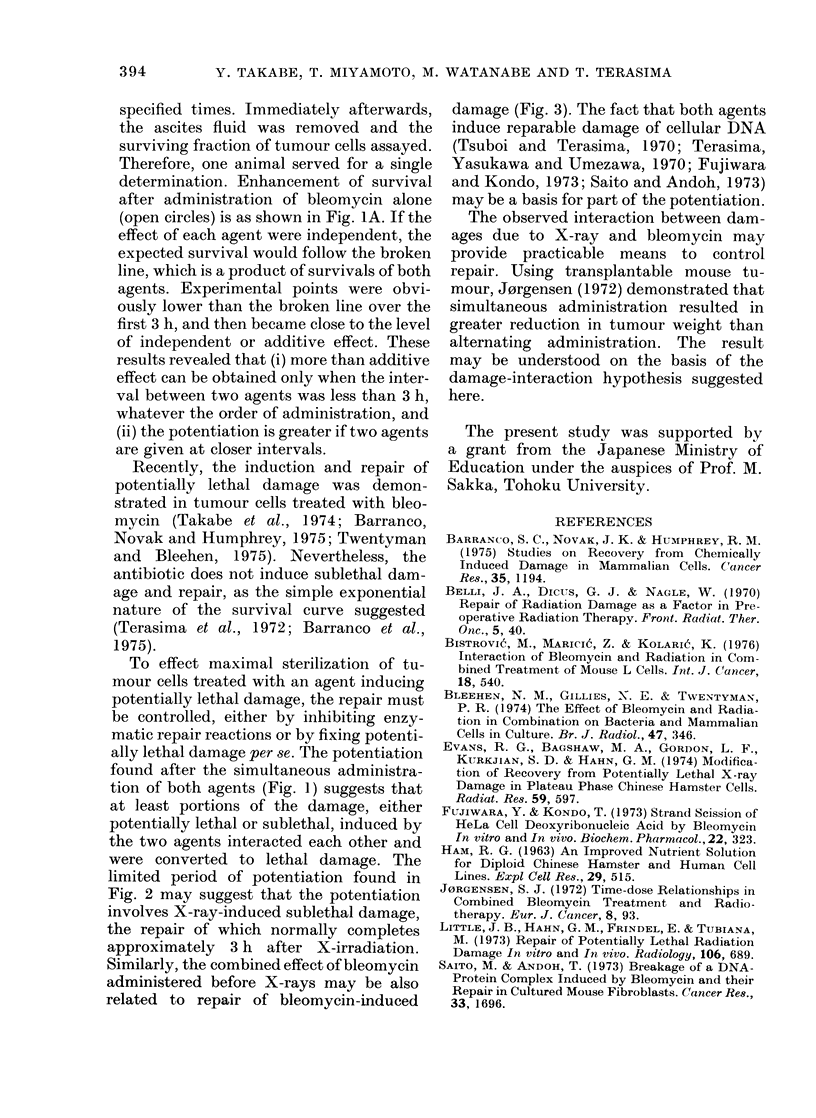

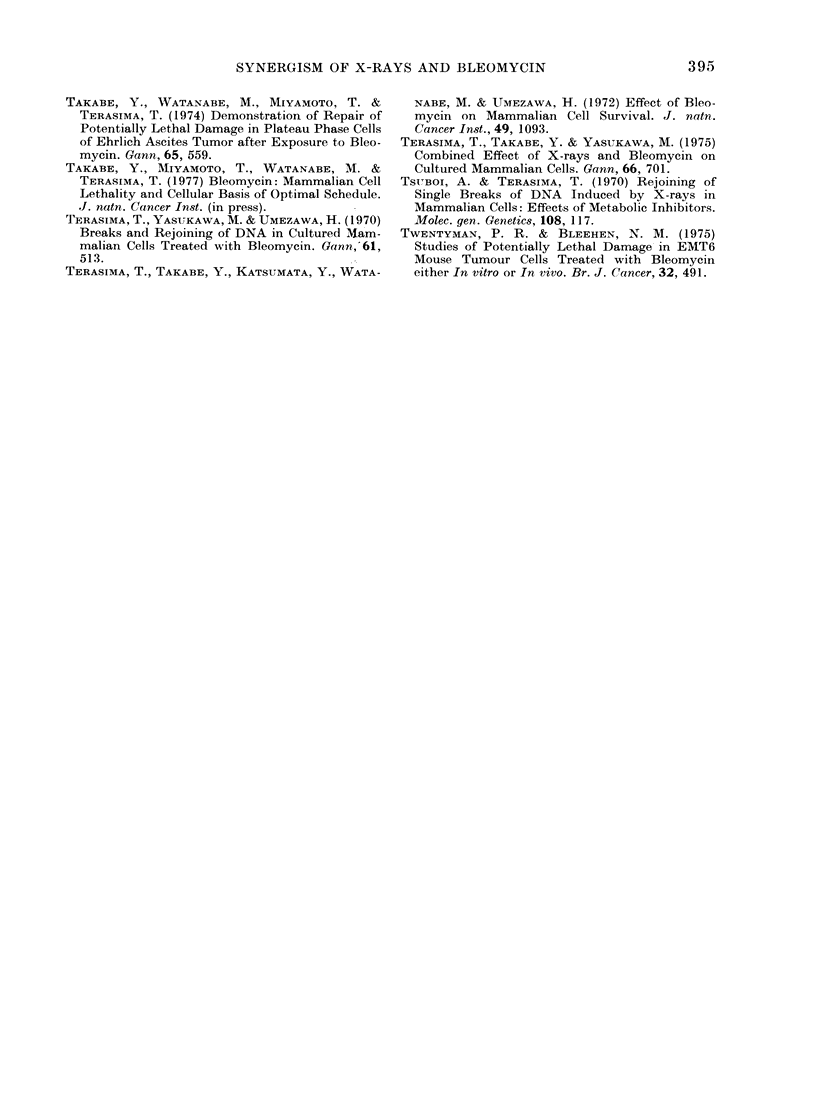


## References

[OCR_00441] Barranco S. C., Novak J. K., Humphrey R. M. (1975). Studies on recovery from chemically induced damage in mammalian cells.. Cancer Res.

[OCR_00453] Bistrović M., Maricić Z., Kolarić K. (1976). Interaction of bleomycin and radiation in combined treatment on mouse L cells.. Int J Cancer.

[OCR_00459] Bleehen N. M., Gillies N. E., Twentyman P. R. (1974). The effect of bleomycin and radiation in combination on bacteria and mammalian cells in culture.. Br J Radiol.

[OCR_00467] Evans R. G., Bagshaw M. A., Gordon L. F., Kurkjian S. D., Hahn G. M. (1974). Modification of recovery from potentially lethal x-ray damage in plateau phase Chinese hamster cells.. Radiat Res.

[OCR_00472] Fujiwara Y., Kondo T. (1973). Strand-scission of HeLa cell deoxyribonucleic acid by bleomycin in vitro and in vivo.. Biochem Pharmacol.

[OCR_00476] HAM R. G. (1963). An improved nutrient solution for diploid Chinese hamster and human cell lines.. Exp Cell Res.

[OCR_00481] Jorgensen S. J. (1972). Dose schedules in bleomycin treatment.. Eur J Cancer.

[OCR_00486] Little J. B., Hahn G. M., Frindel E., Tubiana M. (1973). Repair of potentially lethal radiation damage in vitro and in vivo.. Radiology.

[OCR_00490] Saito M., Ando T. (1973). Breakage of a DNA-protein complex induced by bleomycin and their repair in cultured mouse fibroblasts.. Cancer Res.

[OCR_00498] Takabe Y., Miyamoto T., Terashima T. (1974). Demonstration of repair of potentially lethal damage in plateau phase cells of Ehrlich ascites tumor after exposure to bleomycin.. Gan.

[OCR_00519] Terasima T., Takabe Y., Katsumata T., Watanabe M., Umezawa H. (1972). Effect of bleomycin on mammalian cell survival.. J Natl Cancer Inst.

[OCR_00523] Terasima T., Takabe Y., Yasukawa M. (1975). Combined effect of X-ray and bleomycin on cultured mammalian cells.. Gan.

[OCR_00511] Terasima T., Yasukawa M., Umezawa H. (1970). Breaks and rejoining of DNA in cultured mammalian cells treated with bleomycin.. Gan.

[OCR_00534] Twentyman P. R., Bleehen N. M. (1975). Studies of "potentially lethal damage" in EMT6 mouse tumour cells treated with bleomycin either in vitro or in vivo.. Br J Cancer.

